# Supplementary figures and images for: Choroid structure analysis following initiation of hemodialysis by using swept-source optical coherence tomography in patients with and without diabetes
Source: PLoS One. 2020 Sep 11;15(9):e0239072. doi: 10.1371/journal.pone.0239072 (PMC7485894; doi:10.1371/journal.pone.0239072)

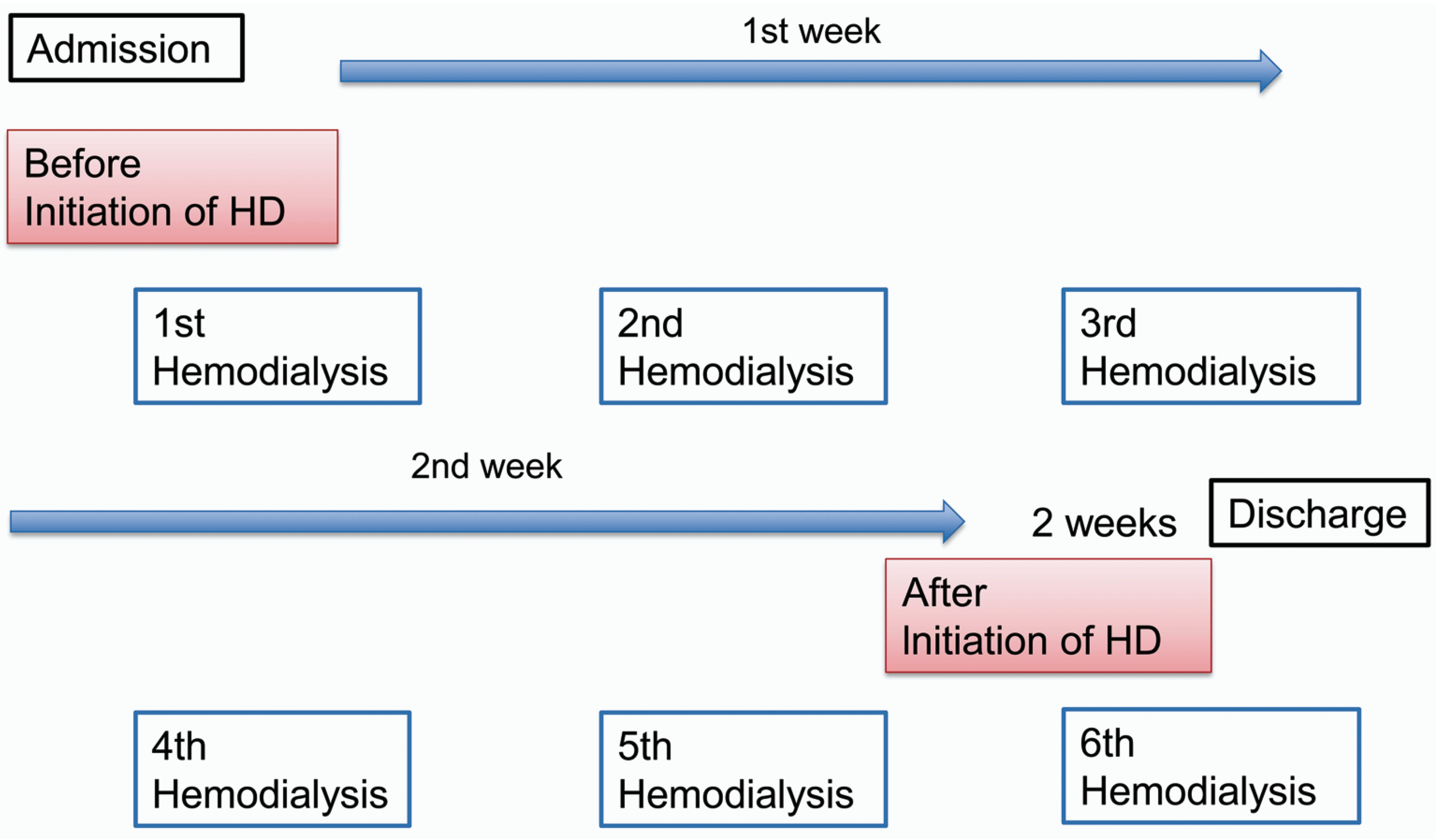

Supplement: S1 Fig — Approximately 6 rounds of hemodialysis were performed during a ~2-week hospital stay. Before the initiation of HD: measurement obtained before the first hemodialysis. After initiation of HD: measurement obtained after the last hemodialysis of the hospital stay. (TIF) [file pone.0239072.s001.tif]
