# Supplementary material for: Choroid structure analysis following initiation of hemodialysis by using swept-source optical coherence tomography in patients with and without diabetes
Source: PLoS One. 2020 Sep 11;15(9):e0239072. doi: 10.1371/journal.pone.0239072 (PMC7485894; doi:10.1371/journal.pone.0239072)
Supplement: S1 Table — (DOCX) [file pone.0239072.s002.docx]

**S1 Table. Changes in systemic parameters before and after the initiation of hemodialysis**

|  | DM group, n = 16 | | NDM group, n = 15 | |
| --- | --- | --- | --- | --- |
|  | Before hemodialysis | After hemodialysis | Before hemodialysis | After hemodialysis |
| Body weight, kg | 70.1 ± 13.9 | 66.5 ± 13.5† | 60.0 ± 11.4 | 57.8 ± 10.5† |
| Heart rate,  beat/min | 75.7 ± 11.1 | 73.5 ± 7.7 | 71.8 ± 9.9 | 67.6 ± 11.3 |
| Blood pressure, mmHg |  |  |  |  |
| Systolic | 155.6 ± 16.0 | 137.1 ± 16.8* | 154.0 ± 21.3 | 137.3 ± 12.6 |
| Diastolic | 76.5 ± 15.4 | 72.6 ± 12.5 | 82.8 ± 8.7 | 74.1 ± 6.2* |
| Mean | 102.9 ± 12.9 | 94.1 ± 12.4† | 106.8 ± 13.9 | 94.5 ± 7.3† |
| Serum osmolarity, mOsm/L | 307.4 ± 7.5 | 299.4 ± 7.7* | 314.6 ± 7.0 | 302.3 ± 6.0† |
| Plasma colloid osmotic pressure, mmHg | 19.9 ± 3.0 | 21.4 ± 2.8* | 21.3 ± 3.9 | 22.0 ± 3.3 |

Before vs. after the initiation of hemodialysis (Wilcoxon signed-rank test); *p < 0.05, †p < 0.01
